# Risk factors associated with xerostomia in haemodialysis patients

**DOI:** 10.4317/medoral.21612

**Published:** 2017-02-04

**Authors:** Rosa-María López-Pintor, Lucía López-Pintor, Elisabeth Casañas, Lorenzo de Arriba, Gonzalo Hernández

**Affiliations:** 1 DDS,PhD, Assistant Professor. Department of Oral Medicine and Surgery, School of Dentistry, Complutense University, Madrid, Spain; 2 Nursing graduate. Haemodialysis Clinic Asyter, Alcázar de San Juan, Spain; 3 DDS,PhD, student. Oral Medicine postgraduate, Department of Oral Medicine and Surgery, School of Dentistry, Complutense University, Madrid, Spain; 4 MD,DDS,PhD, Assistant Professor. Department of Oral Medicine and Surgery, School of Dentistry, Complutense University, Madrid, Spain; 5 MD,DDS,PhD, Professor. Department of Oral Medicine and Surgery, School of Dentistry, Complutense University, Madrid, Spain

## Abstract

**Background:**

To determine the prevalence of xerostomia and hyposalivation in Haemodialysis (HD) patients, to clarify risk factors, assess patient´s quality of life, and to establish a possible correlation among interdialytic weight gain (IDWG) and xerostomia.

**Material and Methods:**

This study was performed on a group of 50 HD patients. Data were collected using a questionnaire containing demographic and clinical variables, a visual analogue scale (VAS) for xerostomia, IDWG, and an oral health impact profile questionnaire (OHIP-14). Unstimulated whole saliva (UWS) and stimulated whole saliva (SWS) were collected.

**Results:**

A total of 28 HD patients (56%) suffered xerostomia. Dry mouth was associated with hypertension (OR, 5.24; 95% CI, 1.11-24.89) and benzodiazepine consumption (OR, 5.96; 95% CI, 1.05-33.99). The mean xerostomia VAS and OHIP-14 scores were 31.74±14.88 and 24.38±11.98, respectively. No significant correlation was observed between IDWG% and VAS and OHIP total score. Nonetheless, a positive correlation between VAS level of thirst and IDWG% was found (r=0.48 *p*=0.0001). UWS and SWS means (determined in 30 patients) were 0.16±0.17 and 1.12±0.64, respectively. Decreased values of UWS and SWS were reported in 53.33% and 36.66% of HD patients.

**Conclusions:**

Xerostomia in HD has a multifactorial aetiology due to accumulative risks as advanced age, systemic disorders, drugs, fluid intake restriction, and salivary parenchymal fibrosis and atrophy. Therefore, it is important to detect possible xerostomia risk factors to treat correctly dry mouth in HD patients and avoid systemic complications.

**Key words:**Haemodialysis patients, xerostomia, salivary flow rate, hyposalivation, interdialytic weight gain, oral health-related quality of life.

## Introduction

End-stage renal disease (ESRD) is the last stage for several primary kidney diseases, and systemic diseases with renal involvement, causing kidney function loss. The most common reasons of ESRD are diabetic nephropathy, chronic glomerulonephritis, interstitial nephritis, hypertension or vascular disease, hereditary or congenital disease and neoplasms (1).

ESRD incidence is increasing, the number of patients being treated for ESRD globally was estimated at 2,786,000 at the end of 2011 and, with a 6-7% growth rate continues to increase at a significantly higher rate than that of the world population. Of these ESRD patients, approximately 1,929,000 were undergoing haemodialysis (HD) treatment (2).

Oral lesions may be observed in HD patients. The most frequent oral disorders observed are xerostomia, hyposalivation, adverse effects related to drug therapy, mucosal lesions as petechiae, gingival hyperplasia, oral infections, dental anomalies and bone lesions (1,3,4).

Xerostomia is a subjective complaint of dry mouth, whereas hyposalivation is an objective decreased of salivary flow. Many cases of xerostomia have been described in patients with a normal salivary flow rate (5,6). Previous studies have shown that the percentage of HD patients who suffer from xerostomia is higher and ranges between 32 and 81% (4,7-13).

In patients undergoing HD, xerostomia is associated with the following problems: difficulties in chewing, swallowing, tasting and speaking (6,14); increased risk of oral diseases, including lesions of mucosa, gingiva and tongue; bacterial and fungal infections, such as candidiasis, dental caries and periodontal disease; interdialytic weight gain (IDWG) resulting from increased fluid intake; and a reduction in quality of life (14).

Xerostomia in HD patients is caused for many factors such as fluid intake restriction, old age, reduced salivary flow, minor salivary glands parenchymal fibrosis and atrophy, mouth breathing and medication use (9,10,12,14,15). Drugs with xerostomizing effects are anticholinergic, sympathomimetic, antihypertensive, cytotoxic, anti-HIV drugs, opiods and benzodiazepines, as well as, anti-migraine agents (6,16). Some psychological factors, such as stress, anxiety or depressive conditions are also related to xerostomia (16,17).

IDWG is a measurable parameter used in the dialysis service to make decisions regarding the amount of fluid removal during a dialysis session. HD patients have to maintain a correct fluid volume balance, which should be achieved by daily restrictions in fluid consumption. Improper drinking behaviours in HD patients lead to fluid overload, which may give rise to hypertension, pulmonary oedema or other cardiovascular manifestations (11,18).

Previous studies have found an important number of HD patients suffered from thirst, xerostomia and saliva reduction (15,18). Likewise, some studies have observed the existence of a positive correlation among thirst, xerostomia and IDWG in HD patients (11,14,18,19). These factors could contribute on morbidity and mortality of HD patients.

The aim of this study is to determine the prevalence of xerostomia and hyposalivation in a group of HD patients, to clarify the risk factors associated to xerostomia in these patients, and to assess patient´s quality or life. The study also tries to establish a connection among IDWG, xerostomia and hyposalivation in HD patients.

## Material and Methods

- Patients

The study was conducted in compliance with the principles of the Declaration of Helsinki. An ethics committee at Hospital San Carlos, Madrid, Spain, approved the study protocol. We obtained a written informed consent from each of the participants prior to his/her inclusion in the study. Patients were included in the study from May 2015 to March 2016. We used the following inclusion criteria with the participants: ≥1 month on HD, 18 years of age or older, mentally and physical ability to participate and complete the study. Patients with hemodynamic instability, therefore preventing them from ultrafiltration, being hospitalized 2-months prior to the study, dementia or terminal diseases, logistic impossibility of investigation, taking any medication or product for his/her dry mouth condition, and/or unwilling to participate in the study were excluded.

We included fifty HD patients in our study and observed them in a haemodialysis clinic, Asyter, in Alcázar de San Juan, Spain. All patients received a fixed HD schedule of 3 times per week. Age, gender, causes of ESRD, underlying diseases, consumed drugs, presence of dentures, tobacco, alcohol, time on HD, dry weight, and body mass index were recorded.

- IDWG Assessment

Each participant was weighed before and after each dialysis session for 2 weeks (6 sessions). IDWG being the amount of fluid (kg) removed during the dialysis session. Dividing IDWG mean of 6 sessions by the patient´s target dry weight and multiplying this result per 100 obtained IDWG%. The target dry weight of the patient was determined according to the standard clinical criteria by nephrologists.

- Xerostomia

The patients that replied positively to the question: “are you normally aware of your dry mouth?,” were considered to have xerostomia (17).

- Xerostomia visual analogue scale (VAS) questionnaire

The patients were questioned about their sensation of dry mouth, and then asked to use a validated VAS questionnaire that contained eight items addressing oral dryness (20). VAS questionnaire was assessed before dialysis session. Subjects were asked to draw a vertical line through a horizontal line, 10 cm long, to indicate their level of dryness for each of the items.

- Assessment of Oral Health Impact Profile (OHIP)-14

The Spanish validated version of the OHIP-14 questionnaire was done to assess patient quality of life (21). It consists of 14 items that assess different aspects of oral function and quality of life. Feedback is based on a Likert format, with a five-point ordinal scale ranging from “never” (coded 1) to “very often” (coded 5). The score ranges from 0 to 70, where higher scores correspond to poorer oral quality of life. The questionnaire was done before dialysis session.

- Saliva collection

Unstimulated whole saliva (UWS) and paraffin chewing-stimulated whole saliva (SWS) were collected before HD session at 8 am. A trained investigator (RMLP), blind to all clinical data and xerostomia and OHIP-14 questionnaires results, collected the saliva. UWS and SWS were only determined in patients that received HD treatment in the morning (30 patients). All subjects were given instructions to not smoke, eat, drink or tooth brush at least 90 minutes prior to saliva collection. UWS and SWS were collected for 15 min using an established spitting technique. The patients were seated in an upright position, and asked to relax during spitting. Saliva volume was determined in millimetres per minute (mL/min). Hyposalivation was present when salivary flow rate was <0.1 mL/min at rest or <0.7 mL/min under stimulation (5,6).

- Statistical analysis

The statistical analysis comprised basic descriptive statistics. The normality of the distribution was checked with the Kolmogrov-Smirnov test. Differences between continuous and categorical variables were assessed by a Student t test, and the chi-square test or Fisher’s exact test, respectively. To explore the association between xerostomia and selected clinical variables, a multiple logistic regression model was fitted. Variables stayed in the model if they were predictors of the outcome (*P*< 0.05). The final model included hypertension, alpha-adrenergic blockers antihypertensives, and benzodiazepines. Pearson´s correlation coefficient was used to assess the correlations between continuous variables. All statistical analyses were conducted using the statistical software SPSS 22.0. Differences were considered significant if *p* was less than 0.05.

## Results

Fifty HD patients (35 men and 15 women) were enrolled in this study. The mean age was 66.62±13.96, and these patients were on HD for 46.02±44.90 months. The clinical characteristics were presented in  Table 1. Twenty-eight patients (56%) were reported to suffer xerostomia. Xerostomia cases showed no significant differences with regard to gender distribution, age, time on HD, causes of ESRD, number of consumed drugs, diabetes, body mass index, dry weight, IDWG average, alcohol and tobacco consumption, and denture presence. Among the xerostomia cases, there were significantly more HD patients with hypertension.

**Table 1:**
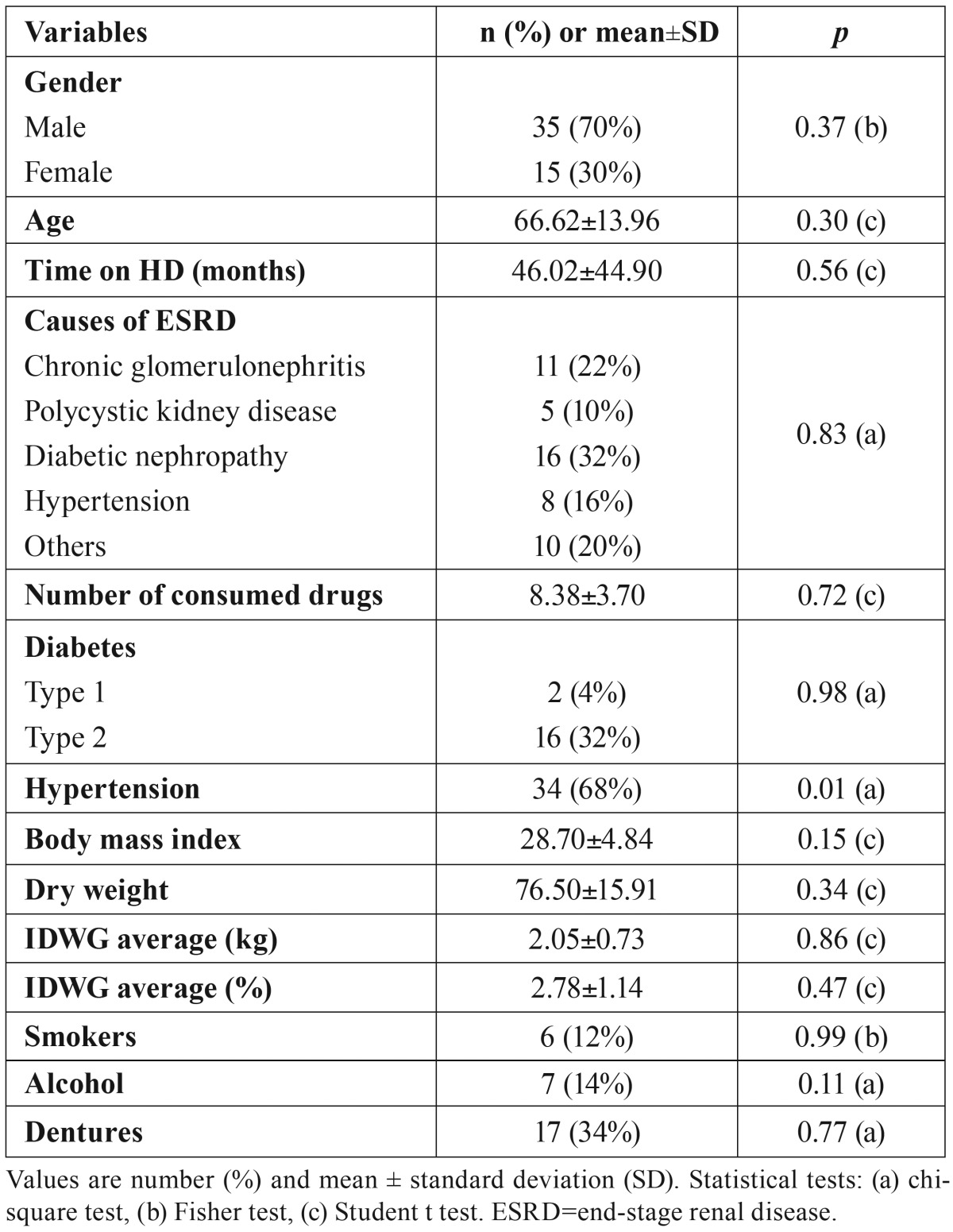
Characteristics of the HD patients and relationship between variables and xerostomia.

Drugs with potential to cause salivary dysfunctions consumed by HD patients of this study were presented in  Table 2. We found statistical correlation between the consumption of alpha-adrenergic blockers antihypertensives and benzodiazepines, and xerostomia sensation.

**Table 2:**
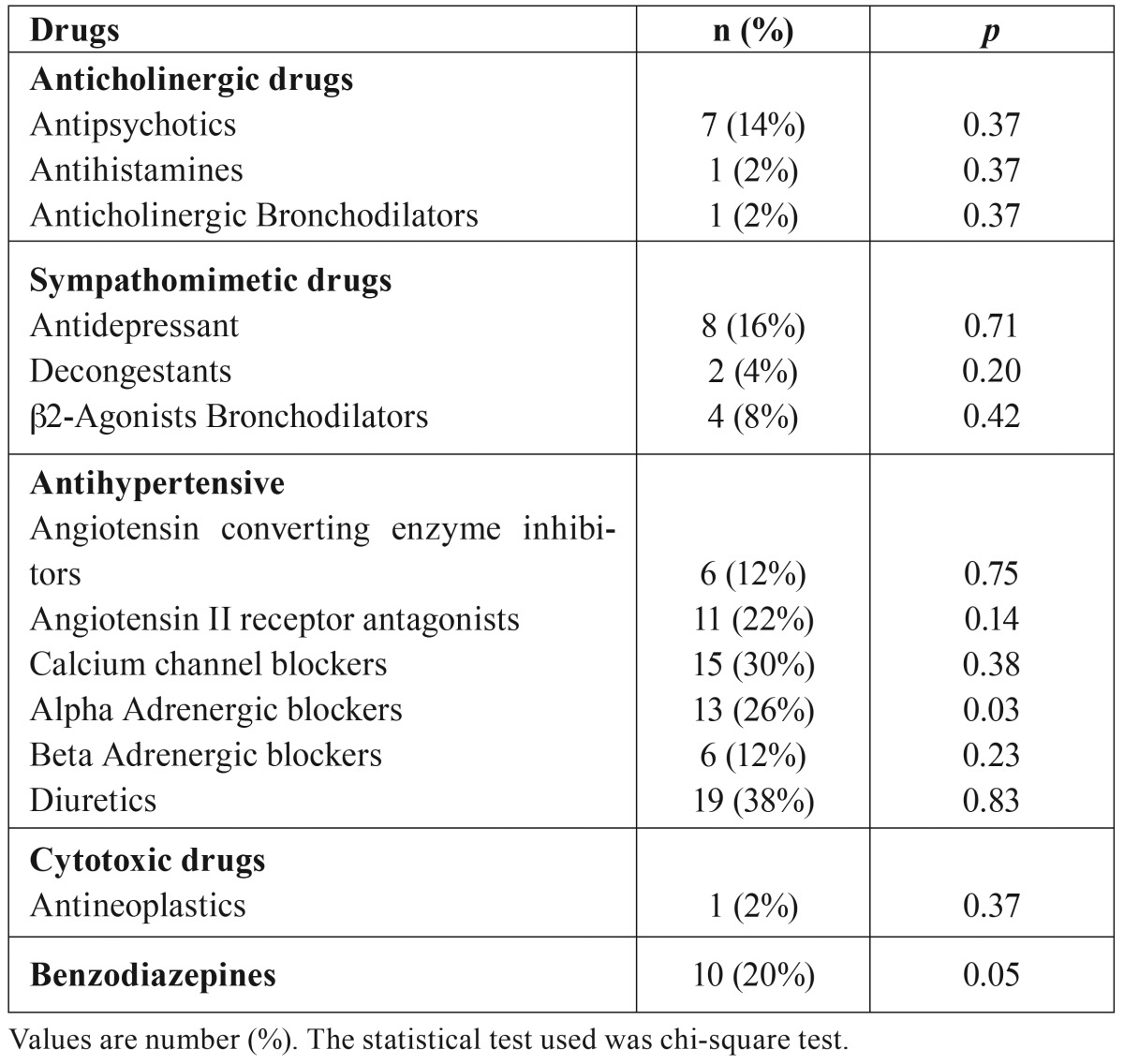
Drugs with potential to cause salivary dysfunctions consumed by HD patients and their relationship with xerostomia.

The final multiple logistic regression model showed odds ratios (ORs) and 95% confidence intervals (CIs). The model showed that xerostomia in HD patients was significantly associated with hypertension (OR, 5.24; 95% CI, 1.11-24.89; *p*=0.03) and benzodiazepine consumption (OR, 5.96; 95% CI, 1.05-33.99; *p*=0.04). Nevertheless, xerostomia was not significantly associated with Alpha Adrenergic blockers antihypertensives consumption (OR, 4.26; 95% CI, 0.94-19.28; *p*=0.06).

The mean VAS score of the study population was 31.74±14.88 ( Table 3), the item 3 (lack of saliva in mouth) and the item 8 (level of thirst) obtained the highest values with 5.45±2.47 vs 5.77±2.78.

**Table 3:**
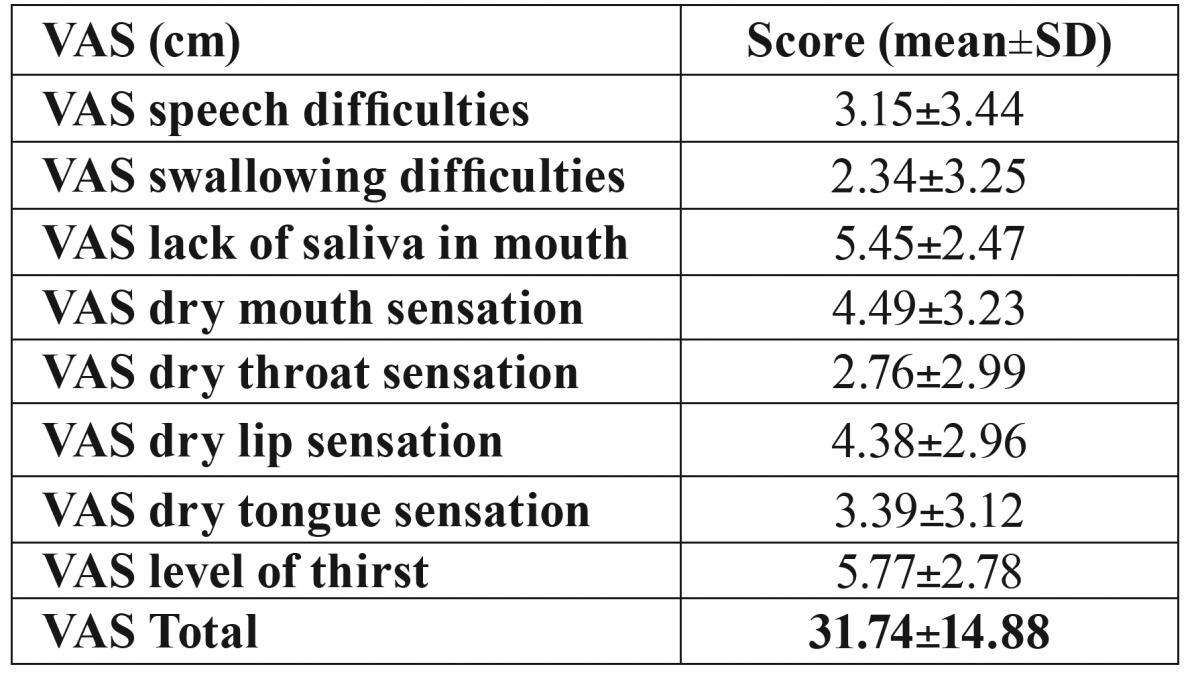
VAS questionnaire scores.

The results of OHIP-14 scores are presented on  Table 4. The mean total score was 24.38±11.98. A positive correlation between xerostomia VAS score and OHIP-14 score was observed in HD patients (r=0.78, *p*=0.0001).

**Table 4:**
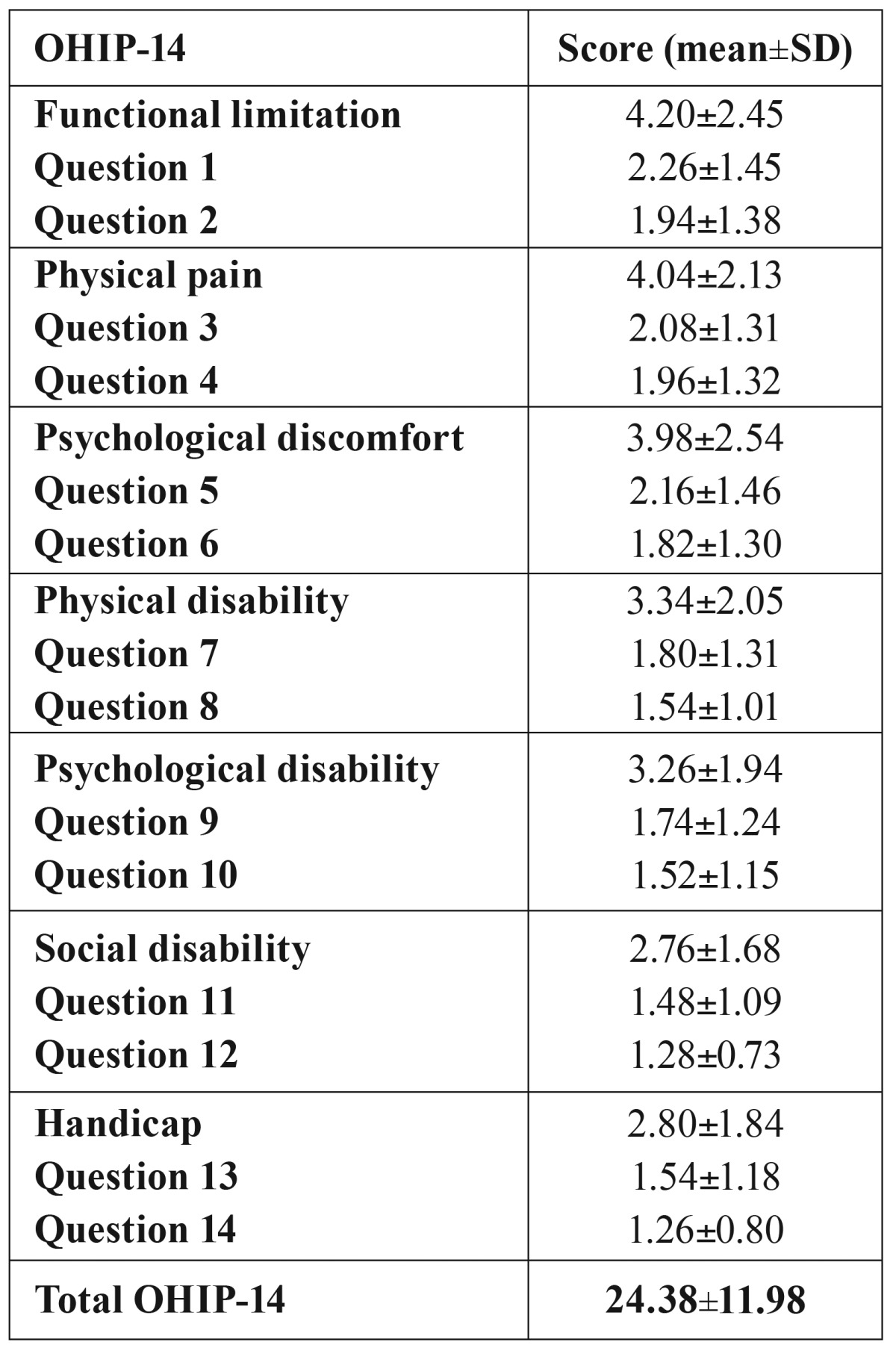
OHIP-14 scores.

We observed that xerostomia HD patients obtained higher VAS and OHIP-14 scores. The mean total VAS score was 38.30±15.07 in xerostomia patients vs. 23.37±9.69 (*p*=0.0001). Mean OHIP-14 scores was 29.64±13.65 in HD patients with xerostomia and 17.68±3.40 in non-xerostomia patients (*p*=0.0001).

No significant correlation was observed between IDWG% and VAS total score (r=0.17 *p*=0.23). Nonetheless, a positive correlation between VAS level of thirst and IDWG% was found (r=0.48 *p*=0.0001). No correlation was noted between OHIP-14 questionnaire and IDWG% (r=0.004 *p*=0.98).

UWS and SWS were only determined in 30 patients that received HD treatment in the morning. The mean UWS and SWS were 0.16±0.17 and 1.12±0.64, respectively. Abnormal values of UWS (<0.1 mL/min) were reported in 16 patients (53.33%). Eleven HD patients (36.66%) had abnormal levels of SWS (<0.7 mL/min).

Of 30 patients, 57.1% of HD with UWS <0.1 mL/min and 33.3% of HD with SWS <0.7 mL/min had xerostomia (*p*=0.69 vs. *p*=0.68). We did not find a relationship between the volume of UWS and SWS, and xerostomia (*p*=0.51 vs. *p*=0.53).

Negative correlations were found between UWS (r=-0,25, *p*=0.18) and SWS (r=-0.14, *p*=0.46) and the total OHIP-14 score, patients with higher OHIP-14 levels had lower volumes of saliva, but these correlations were not significant. Nevertheless, a significant negative correlation was found between UWS and VAS score (r=-0.46, *p*=0.01), patients with higher VAS score had lower volume of saliva. No significant correlation between SWS and VAS score (r=-0.30, *p*=0.11) was observed.

## Discussion

The prevalence of xerostomia varies from 6 to 30% in older European adult population (11,14). Previous studies found that xerostomia was the most common oral disorder in HD patients (22), and showed that percentage of HD patients who suffer from xerostomia is higher, and ranges between 32 and 81% (4,8-13,19). The results of this study show a xerostomia prevalence of 56% in HD patients, our results are in accordance with the range prevalence found previously in HD patients, and it is higher than prevalence found in general older population.

It is known that main factors associated with xerostomia are ageing, head and neck radiotherapy, systemic disorders, and several drugs. Systemic diseases associated to xerostomia are rheumatological chronic inflammatory disorders (Sjögren syndrome, rheumatoid arthritis, systemic lupus erythematosus), endocrine disorders (diabetes mellitus, hyperthyroidism and hypothyroidism), neurologic disorders (depression and Parkinson´s disease), genetic disorders, metabolic disorders (dehydration, anaemia and alcohol abuse), infectious disorders (HIV/AIDS, HCV infection), and others (fibromyalgia, sarcoidosis and chronic pancreatitis) (6,23). A lot of cases of xerostomia are related to psychological conditions like depression and anxiety (16,17).

Different papers have studied the effects of ageing on salivary production, but there is still controversy with regards to salivary dysfunctions in the elderly. There are studies that have demonstrated impaired glandular function, and others have not found salivary dysfunctions among healthy, non-drug consuming, aging population (6,23). In HD patients, there are studies that have shown that dry mouth is associated with age (18,19,24). Nevertheless, we have not found a relationship between age and xerostomia in our patients.

Most HD patients have complex medical conditions, including hypertension and diabetes, and a lot of HD patients take many medications that increase the risk of xerostomia (4). In this study, we recorded the systemic diseases suffered by each HD patient, and observed a relationship between hypertension and xerostomia. It is difficult to clarify if xerostomia is associated with high blood pressure or to hypertension treatment that is related to dry mouth, as we can see below.

It is not clear if diabetic HD patients have higher xerostomia prevalence. Diabetes mellitus and disturbances in glycaemic control could damage the gland´s parenchyma, and cause alterations in the micro-circulation of the salivary glands as dehydration (6). There are studies that have shown that xerostomia was severe in diabetic compared with non-diabetic HD patients, and HD patients with poor glycemic control also showed higher xerostomia incidence (25). In addition, there are studies that observed that HD patients that begin HD due to diabetic nephropathy have greater xerostomia than those that begin due to glomerulonephritis (1). Although, authors like Swapna *et al.* (13) did not observe a significant relationship between xerostomia and diabetes among HD patients. We, as well, have not found a relationship between diabetes and xerostomia but we think that it is necessary further research to clarify a possible influence of diabetes on dry mouth in HD patients reflecting also their glycaemic levels.

As we described earlier, there are lots of drugs with potential to cause salivary dysfunction. Unfortunately, little data about the effects of many supposed xerostomia-inducing drugs on salivation are available (6). According to some authors, xerostomia prevalence rises with increasing numbers of drugs used (23,26). In the present study, it was observed that the number of consumed drugs did not increase the risk of suffering xerostomia. Although, we found that among the different drug categories associated with xerostomia taken by HD patients, only alpha-adrenergic blockers and benzodiazepines were associated with dry mouth.

Dry mouth has been related as a side effect of different kinds of drugs for antihypertensive therapies, which produce salivary dysfunction in different ways (6,16). Diuretics cause an overall decrease in intravascular and extracellular fluid volume and, as a consequence, decrease salivary flow rate (6). Anti-hypertensives that act in alpha-2-adrenergic receptors frequently cause xerostomia. There are authors that have demonstrated that alpha 2-adrenergic receptors activated alpha 2-adrenoceptor in the lateral hypothalamus, and this activation has anti-salivatory effects. Calcium channel blockers depress calcium influx due to inhibition of the voltage-dependent calcium channels, thereby decreasing the acetylcholine-induced calcium elevation, and produce an inhibition of salivation (6). Angiotensin-converting enzyme inhibitors, alpha and beta-adrenergic blockers, and angiotensin II receptor antagonists are also related to xerostomia (26). In this study, we observed that hypertensive HD patients have an increased risk of suffering xerostomia vs. non-hypertensive HD patients. Nevertheless, we cannot compare our results with other papers because there are no studies about xerostomia in HD patients regarding the subject.

Benzodiazepines decrease the salivary flow rate through the benzodiazepine receptors in the salivary glands and by indirect action on the salivary glands through the central benzodiazepine receptors (27). In this study, we observed an increased risk of dry mouth in HD patients taking benzodiazepines. As mentioned before, we have not found papers discussing HD patients.

There are studies that have evaluated the intensity of xerostomia in HD patients. Different subjective test and questionnaires have been used, VAS for thirst (only one item), VAS for xerostomia (only one item), dialysis thirst inventory and xerostomia inventory. Previous studies have shown a xerostomia inventory that ranges from 20.6 to 33.1 in HD patients (1,11,19). The xerostomia inventory includes 11 items, each with a 5-points scale, the results range from 11 (no dry mouth) to 55 (extremely dry mouth) (15). We assessed the rate of xerostomia by VAS questionnaire, validated to evaluate the level of dry mouth (6,20). In our study, the mean VAS score of the HD patients was 31.74±14.88. Although xerostomia inventory and VAS values are no comparable, it seems that our results are lower than previously.

In this study, we used the validated OHIP-14 questionnaire (21) to assess patient’s quality of life. We obtained a mean total OHIP-14 score of 24.38±11.98, and a positive correlation between VAS score and OHIP-14 score were observed in HD patients. Also, our mean OHIP-14 scores were significantly higher in xerostomia patients. There are no previous studies that have evaluated OHIP-14 in HD patients, but other authors (18) have used other quality of life questionnaires related to kidney disease like kidney disease quality of life (KDQOL). Fan *et al.* (18) observed that xerostomia is related to higher values of KDQOL. Our results cannot be compared with this study because the quality of life questionnaire was different, but we can conclude like Fan *et al.* (18) that xerostomia get worse the quality of life of HD patients. Therefore, treating xerostomia in HD patients could improve their quality of life.

There are studies (7,11,18,19) that have evaluated if xerostomia or thirst sensation were related to IDWG. Some studies have observed a significant positive correlation between xerostomia, thirst and IDWG (11,19), HD patients with high levels of thirst, and xerostomia gained more weight between HD sessions. However, there are papers (28) that have not found a significant correlation between IDWG and thirst intensity. In this study, we found a positive, non-significant correlation between IDWG% and total score of xerostomia VAS questionnaire, and a significant positive correlation between VAS level of thirst and IDWG%. We think, like other authors (11), that a thirst sensation produces a frequent intake of fluids, HD patients could refuse to comply with restrictions on fluids due to thirst sensation, which could enhance IDWG in HD patients. Overall, this problem could be very complex due to possible multifactorial aetiology and the physiology is not known.

The sensation of xerostomia could be associated to hyposalivation, but this association is not always present (29). In our study, we only determined salivary flow in 30 HD patients that received HD treatment first thing in the morning due to the influence of time measurement of saliva on the diagnosis of hyposalivation. The saliva test was performed at a fixed time-point of a limited time interval early morning due to the circadian rhythm of salivary flow (30). There are studies that showed that UWS flow rate varies between 0.4±0.3 and 0.45±0.25 mL/min in older non-HD patients (9,29). The UWS mean of previous HD studies varies between 0.28±0.16 and 0.31±0.28 mL/min (8,9,11,19). Our UWS mean (0.16±0.17) was lower than aforementioned reports.

According to recent criteria, hyposalivation can be defined as UWS rates below 0.1 mL per/min and SWS rates below 0.7 mL per/min (6,29), and appeared in 12.1% in the general population (29). Previous studies have found UWS hyposalivation in 28.8-40.5% of HD patients (11,18,19). In our study, hyposalivation was greater than previous general population and HD studies. We obtained hyposalivation UWS and SWS values in 53.33% and 36.66% of HD patients, respectively. We found that 57.1% of HD with UWS <0.1 mL/min and 33.3% of HD with SWS <0.7 mL/min had xerostomia; therefore, we have not found relationship between hyposalivation and xerostomia. We think that these discrepancies in mean UWS flow rate and hyposalivation prevalence could be due to different criteria to define hyposalivation (sometimes <0.15ml/min flow rate) and time of collection, is not specified in the previous papers, that we saw previously could influence in the quantity of salivary flow.

In conclusion, xerostomia is a frequent problem in HD patients and it is related to many accumulative xerostomia risks in these patients (ageing, systemic disorders, drugs, fluid intake restriction, and salivary parenchymal fibrosis and atrophy). In this study, hypertension and benzodiacepine use was related to this problem. So, to treat xerostomia correctly in this group of patients, we have to take into consideration all potential factors related to dry mouth. It is not clear if xerostomia could influence the increase of IDWG. We believe that it is necessary to realize studies with a great number of HD patients to justify this possible relationship. In this study, we have confirmed that the hyposalivation it is not always present in xerostomia HD patients, thus could be an open door to associate xerostomia in HD patients to possible psychological problems.
